# Bacterial Profile and Their Antimicrobial Susceptibility Pattern of Isolates Recovered from Intensive Care Unit Environments at Wachemo University Nigist Ellen Mohammed Memorial Comprehensive Specialized Hospital, Southern Ethiopia

**DOI:** 10.1155/2023/1216553

**Published:** 2023-09-15

**Authors:** Muluneh Temesgen, Abera Kumalo, Takele Teklu, Getachew Alemu, Desta Odoko

**Affiliations:** ^1^Department of Medical Laboratory Science, Hosanna Health Science College, Hosanna, Ethiopia; ^2^Department of Medical Laboratory Science, College of Health Sciences and Medicine, Wolaita Sodo University, Sodo, Ethiopia; ^3^Department of Medical Laboratory Science, Sodo Christian General Hospital, Sodo, Ethiopia

## Abstract

**Background:**

Bacterial contamination of indoor hospitals, especially in intensive care units is a serious health hazard in the world with a high morbidity and mortality rates. Particularly, multidrug-resistant bacteria can cross-contaminate medical devices, inanimate surfaces, health care providers, and patients in the intensive care unit. This study was aimed to assess the bacterial profile and their antimicrobial susceptibility patterns of bacterial isolates from intensive care unit at WUNEMMCSH (Wachemo University Nigest Ellen Mohammed Memorial Compressive Specialized Hospital), Southern Ethiopia.

**Methods:**

A hospital-based cross sectional study was conducted on 180 intensive care unit environmental samples at WUNEMMCSH from August 1, 2022, to October 30, 2022. In this study, a total of 180 swab samples were collected from medical devices, inanimate surfaces, patients, and health care providers from the intensive care unit by using sterile cotton-tipped swabs moistened with normal saline. Then, bacterial isolates were identified using the standard culture method, Gram stain, and biochemical tests. Antimicrobial susceptibility tests for bacterial isolates were performed by using the Kirby Bauer disk diffusion method. Data were entered into EpiData Version 4.6 cleanup and exported to SPSS V25 for analysis.

**Results:**

A total of 180 swab samples were processed from intensive care unit environments, and 143 (79.4%) were found to have been contaminated by at least one potential pathogenic bacterial isolate. A total of two hundred and thirty-eight bacteria were isolated. Of these, the predominant bacteria were coagulase-negative *Staphylococci* 72 (30.3%), *S. aureus* 61 (25.6%), *E*. *coli* 41 (17.2%), and *K. pneumoniae* 30 (12.6%). Seventy (49%) out of all swabbed samples were contaminated with mixed isolates. In the antimicrobial susceptibility tests, 19 (86%) Gram-positive bacteria and 25 (76%) Gram-negative bacterial isolates were susceptible to nitrofurantoin, respectively. Vancomycin was sensitive to 83% of Gram-positive isolates. Gram-positive and Gram-negative isolates from irrespective sources showed multidrug resistance in 56.4% and 76.2%, respectively.

**Conclusion:**

The inanimate hospital environments, medical device, health care providers, and patients in ICU rooms of WUNEMMCSH (Wachemo University Nigest Ellen Mohammed Memorial Comprehensive Specialized Hospital) were colonized with 143 (79.4%) of potential pathogenic bacterial isolate, which can cause nosocomial infections with high rates of morbidity and mortality among patients. The frequencies of multidrug-resistant 159 (66.8%) bacterial pathogens were alarmingly high. Therefore, to reduce the risk of bacterial contamination and MDR, strict adherence to hospital infection prevention and control measures should be enforced. These measures include regularly performing hand hygiene, periodic disinfection, and sterilization of medical equipment.

## 1. Introduction

The intensive care unit (ICU) is one of the leading units among health care settings in the percentage of the population with nosocomial infections [1] because of its patients who are susceptible to various infections due to immunodeficiency, utilization of invasive medical equipment, administration of various medications, or deformation of normal flora. Health care-associated infection (HCAI) is an infection acquired recently by patients who have been admitted to a health care facility for the purpose of other health care needs. It can happen two to three days after admission or one month or more after release from admission. It results in mortality, morbidity, and expenses, as well as an increase in the hospital stay [2]. Infections acquired in intensive care units (ICUs) are a serious health hazard in the world with high morbidity and mortality rates, especially due to cross-transmission of germs [3].

Being frequently coined reservoirs of multidrug resistant (MDR) pathogens, the intensive care unit (ICU) inanimate environment has warranted much attention for its probable contribution to thetransmission of nosocomial infections [4] and may serve as potential reservoirs for nosocomial infection and facilitate transmissions through contact, depending on their tenacity [5, 6], and according to the World Health Organization (WHO) [7]. Contamination can occur as a result of health care professionals and patient hands or direct shedding of microorganisms that can live for long periods on dry surfaces [8].

Many microorganisms have the potential to cause infections in hospitalized patients [9] because their immunity may be compromised, making them more susceptible to infection. Bacterial pathogens such as coagulase-negative staphylococci (CoNS), *Enterobacteriaceae*, *S. aureus*, *Pseudomonas* spp., *Haemophilus influenzae, Klebsiella pneumoniae, Enterococci,* and *Acinetobacter* spp. are the most common microbe responsible for approximately 90% of HAIs, and they can be found in patients, health care workers, attendants, contaminated instruments, and the environment which are able to survive and persist for long periods of time in the hospital environment [10]. They also have resistance potential for disinfectants, while protozoa, fungi, and viruses are less attributed to nosocomial infection [11].

Approximately 2 million hospitalized patients in the United States have clinically severe nosocomial infections, resulting in an annual increase in costs [12]. Patients who are admitted in the ICU are at a high risk of contracting nosocomial infections as a result of breaches in host defense caused by trauma, invasive medical devices, and/or corticosteroid medication [13].

The ability of bacteria to develop resistance to antimicrobial agents has made treating bacterial infections more difficult in recent years. The emergence of resistance to antimicrobial agents is a global public health problem, particularly in pathogens causing nosocomial infections which contributed to the morbidity, mortality, increased health care costs resulting from treatment failures, and longer hospital stays from invasive procedures, high antibiotic usage, and transmission of bacteria among patients due to inadequate infection control measures explain why ICUs are “hot zones” for the spread of antibiotic resistant organisms [14]. Also, most of the admitted patients in ICU are treated with empirical prescribed antibiotics, but it commonly leads to antimicrobial resistance (AMR) [15] and/or emergence of multidrug resistance (MDR) as well as death of patients [16, 17]. The emergence of MDR strains in the ICU of hospitals, particularly in developing countries, is an increasing problem that is posing a barrier to the management of HCAIs [18] due to high patient fellow, which is a critical role played by the inanimate environment in the transmission of nosocomial infections. Furthermore, antibiotic resistance can be introduced into the ICU invisibly and silently by a commensal member of the patient's or health care personnel's microbiome [19].

The problem may be further complicated in this study area due to lack of advanced laboratory diagnostics and higher rate of inappropriate empirical antibiotics treatment, cross-contamination between equipment, high patient flow, and health care workers. The drug susceptibility pattern of the isolates to commonly used antimicrobials in this area will also provide vast options for clinicians to select appropriate antimicrobials for empirical therapy. So this study was aimed at the isolation and antibiotic susceptibility pattern of potentially pathogenic bacterial isolates at ICU environments of the hospital setup.

## 2. Methods

### 2.1. Study Area

The study was conducted in Wachemo University Nigest Ellen Mohammed Memorial Comprehensive Specialized Hospital, which is located in Hosanna, Hadiya Zone, Southern Nations, Nationalities and People's Regional state. The town is 232 kilometers from Addis Ababa, the capital of Ethiopia. This hospital was established in 1984 E.C in order to serve the catchment population of about 2.5 million in the zone and nearby zones and districts. The service provided by these hospitals includes the following: surgery, pediatrics, internal medicine, outpatient diagnosis, inpatient, operation room, gynecology, obstetric emergency, antenatal care, family planning, intensive care unit service, tuberculosis clinic, psychiatry, physiotherapy, dental, ophthalmology, laboratory, pharmacy, cervical cancer prevention, radiology, and health education. Wachemo University Nigest Ellen Mohammed Memorial Comprehensive Specialized Hospital has around 300 beds, and it is a center of excellence for training of undergraduate and postgraduate students in different health-related disciplines. The hospital ICU (intensive care unit) is staffed with around 100 health care providers of different qualifications. The hospital data show that the ICU has 65 beds giving admission care for around 61 patients per month in average.

### 2.2. Study Population, Study Design, and Study Period

A hospital-based cross sectional study was conducted at ICU environments (inanimate objects, medical devise, health care providers, and patients who were admitted to ICU) during the study period from August 1, 2022 to October 30, 2022.

### 2.3. Sample Size Determination

Sample size was determined using single population proportion formula considering the following assumptions: 95% confidence interval, 5% margin of error, and the prevalence (*P*) of assumed prevalence of 87.3% from the previously conducted study on medical devices and inanimate surfaces in Bahir Dar [20].

The following standard formula was used to calculate it:

(1) $$\begin{array}{c} \text{The sample size }n=\frac{Z\alpha^{2}\;P\;\left(1-P\right)}{d^{2}}\text{,}\\ Z\left(\frac{\alpha }{22}\right)=\text{at }95\%\text{ confidence interval }Z\text{ value }\left(\alpha =0.05\right)=1.96\text{,}\\ p=\text{Prevalence of previous study }87.3\%\;\left(0.873\right)\text{,}\\ d=\text{Margin of error at }\left(5\%\right)\left(0.05\right)\text{,}\\ n=\frac{\left(1.96\right)2\left(0.873\right)\left(1-0.873\right)}{{\left(0.05\right)}^{2}}=170\text{.} \end{array}$$

According to information obtained from Wachemo University Nigest Ellen Mohammed Memorial Comprehensive Specialized Hospital, 61 patients were admitted per month on average, and in the three-month period that was estimated to 183 in ICU, the total health care providers were 100 and there were a total of 125 medical devices and 94 inanimate surfaces, respectively, found in the ICU room that were functional during data collection period. For each study individual and environmental surfaces, the sample size was calculated by using proportional allocation by formula, ni = Ni/N ∗ n. Then, the calculated sample size for medical devices was 43, and for inanimate surfaces 32. By considering a 10% of nonrespondent rates for health care workers and patients, the sample size of health care workers was adjusted to 37, and for patients 68. Hence, the total sample size for the study was 180.

### 2.4. Sampling Techniques and Data Collection

The study participants and environmental swab samples were selected from ICU room by convenient sampling technique until the required sample size was achieved. A swab sample was collected from medical devices, inanimate surfaces, hands of patients, and health care providers after 2 hours of disinfection of the ICU rooms at daytime, considering the most representative hours (at 8:00 AM in the morning and 2:00 PM in the afternoon) after a preliminary survey by considering the fact that a higher load of patients with its attendants, clinicians, and medical devices used by them become burden for acquiring microorganisms through close contacting in ICU environments of the hospital and 10 cm^2^ regions were swabbed to ensure uniform sampling. The surface areas of tiny objects or surfaces were approximated and the entire region was swabbed, emphasizing the frequently touched areas in two directions at right angles to each other in a close zigzag pattern at each site, rotating the swab during sampling to ensure that the full surface of the swab is utilized. Most frequently touched part in inanimate or medical device surface swab and most representative locations in each study participants (health care providers and patients) were selected to sample and the sampling sites were categorized into four groups: (1) commonly touched medical devices including stethoscopes (ear piece, tubing, and diagram), sphygmomanometer (bulb and cuff), body incubator (sides and top), suction machine (tubes and needle), weighing scale (sides and top), and thermometer NICU (mercury and tubes). (2) Commonly touched inanimate surfaces including patient bedside surfaces (bedside table bottom and bedside table top), wall surfaces (close contact to patient), door handle, table (top and sides of table), and chair (sides of chair). (3) Hands of patients (fingers and palm area of the hands). (4) Hands of health care providers (fingers and palm area of the hands) [21].

### 2.5. Sample Collection and Transportation

Self-contamination was prevented by wearing sterile disposable gloves, mouth masks, and protective gown. The swab samples were employed to collect from inanimate, medical device, and study individuals. Prelabeled sterile cotton swab sticks were moistened in 0.85% sterile normal saline and were rolled over the medical devices and inanimate surfaces, hands of patients, and health care providers (each 10 cm^2^ surface area) [21]. Each swab was aseptically replaced into a test tube containing 10 ml of normal saline and sealed. Then, they were properly labeled and all collected specimens were transported to the microbiology laboratory of Southern Nations, Nationalities, and People's Region Health Bureau, Hosanna Branch Regional Laboratory Institute within 30 minutes to one hour, and the solution containing the swab was thoroughly agitated with a vortex mixer to release the bacteria from the swab. Then, all swab samples from the pieces of equipment and each study individuals were analyzed by clinical laboratory standard protocols [22].

### 2.6. Processing of Specimens and Preliminary Identification

Isolation and identification of bacteria were performed by clinical microbiology techniques such as the culture method, Gram stain, and biochemical tests. A 100 *μ*l of the all diluted sample was aseptically inoculated using a sterile spreader onto each of 5 percent sheep blood agar plates (Oxoid Ltd., UK) for both Gram-negative and Gram-positive bacterial isolates, MacConkey agar (Oxoid Ltd., UK) for Gram-negative bacterial isolates, and mannitol salt agar (Oxoid Ltd., UK) for identification of *Staphylococcus* species. The inoculated plates were incubated at 37°C for 24–48 hours and observed for any bacterial growth. Subcultures of the respective bacterial isolates were subsequently subjected to species identification and confirmation for the initial screening of suspected pathogens and an estimation of the colony characteristics. The grown colonies' characteristics of isolated bacteria have been identified by Gram staining and standard biochemical tests. Gram-positive cocci were identified by Gram staining from blood agar culture media. Also, they were identified using catalase and coagulase tests from mannitol salt agar. Members of *Enterobacteriaceae* family were identified by a series of biochemical tests including the following: catalase, indole, citrate, motility, urease, H2S production, and oxidase test and triple-sugar iron. Nonlactose fermenting Gram-negative bacteria were identified by indole, triple-sugar iron, urease, oxidase, motility, and catalase tests. If there was no sign of growth after 48 hrs of incubation, the sample was reported to be culture negative [22].

### 2.7. Antimicrobial Susceptibility Testing

Antibiotics were selected based on local availability, information from literature, and effectiveness. Antimicrobial susceptibility patterns of the bacterial isolate were tested using the modified Kirby–Bauer disk diffusion method on the Mueller–Hinton agar (MHA) (Oxoid Ltd., UK) according to the Clinical Laboratory Standard Institute guidelines [23]. The colonies were emulsified in a sterile normal saline solution, and densities of suspension were compared with the opacity standard on a 0.5 McFarland solution to give a density equivalent to that of the standards [24]. A sterile swab was dipped into the suspension of the isolate in broth; excess fluid was removed against the side of the bottle. The entire surface of the MHA plate was swabbed with the test organism suspension, turning the plate 360 degrees and repeating the process three times. The antimicrobial disks were placed on the surface of the agar and gently pressed down with sterile forceps. Then, the medium was incubated at 37° C for 18–24 hours. The results were reported as sensitive, moderate, or resistant using the Clinical and Laboratory Standards Institute's (CLSI) breakpoints [25]. Antibiotic disks containing methicillin 5 *μ*g, cloxacillin 5 *μ*g, nitrofurantoin 300 *μ*g, tetracycline 30 *μ*g, cefoxitin 30 *μ*g, gentamicin 30 *μ*g, azithromycin 15 *μ*g, cefixime 5 *μ*g, ciprofloxacin 5 *μ*g, clindamycin 10 *μ*g, chloramphenicol 30 *μ*g, vancomycin 30 *μ*g, meropenem 10 *μ*g, ceftazidime 30 *μ*g, cotrimoxazole 25 *μ*g, ceftriaxone 30 *μ*g, ampicillin 10 *μ*g, and amoxicillin/clavulanic acid 10 *μ*g were used [26].

### 2.8. Operational Definitions

- Load: it is the number and type of microorganisms contaminating medical equipment or human being.
- Contamination: it is the state of containing unwanted microorganisms or substances in land or groundwater that are or potentially hazardous to the hospital environments or human health.
- Highly antibiotic resistant: they are microorganisms that are not controlled or killed by antimicrobials. They are able to survive and even multiply in the presence of antimicrobials.
- Inanimate surface: it is the type of not animate equipment used in the ICU for giving patient health care such as beds, tables, mattresses, door handle, and wall surfaces.
- Medical equipment: equipment used for the management of patients admitted to the ICU. They include such items as stethoscope, sphygmomanometer, and monitors.
- Multidrug resistance (MDR): it is the antibiotic resistance ability of bacteria, i.e., resistance to greater than one antibacterial drug in three or more antibiotic classes.

### 2.9. Data Quality Control

The reliability of the study results was guaranteed by implementing quality control (QC) measures throughout the whole processes of the laboratory works. Aseptic techniques were observed in all the steps of specimen collection and inoculation onto culture media to minimize contamination. All culture media have been prepared according to the criteria of the manufacturers. Culture media were tested for sterility and performance. The standard operating procedures (SOPs) were strictly followed during each laboratory work. Control bacteria strains such as *Escherichia coli* (ATCC 25922), *S. aureus* (ATCC 25923), and *Pseudomonas aeruginosa* (ATCC 27853) were used in controlling the potency of the drugs.

### 2.10. Data Analysis

Data were coded and entered into EpiData 4.6, and then it was transported to Statistical Package for the Social Sciences (SPSS) version 25 that was used to analyze the work and to make inferences on the frequency of occurrence. A frequency analysis such as descriptive statistics (tables, graphs, and charts) has been carried out to determine the general status of study.

### 2.11. Ethical Considerations

Ethical clearance was obtained from WSU Health Research Ethics Review Board. A formal letter of cooperation was written to Wachemo University Nigist Ellen Mohammed Memorial Comprehensive Specialized Hospital. Permission was obtained from the hospital to conduct the study. For data collection, informed consent was obtained from health care providers and admitted patients after explaining the purpose and procedure of the study. All information obtained from the study participants were secured and coded to maintain confidentially. Disclosure of laboratory findings to Wachemo University Nigest Ellen Mohammed Memorial Comprehensive Specialized Hospital was made.

## 3. Results

### 3.1. Bacterial Isolates from ICU Environments

A total of 180 swab samples were collected from medical devices, inanimate surfaces, patients, and health care providers in the ICU environments. Of these, 68 (37.8%) were from patients hand, 37 (20.5%) were from health care providers, 43 (23.9%) were from medical devices, and 32 (17.8%) were from inanimate surfaces with the total response rate of 100% by swabbing. Out of 180 swabs, 143 (79.5%) swabs were contaminated by at least one bacterium isolates, of which 54 (37.5%) were from patients in ICU, 24 (16.5%) were from health care providers working in ICU, and 37 (26%) were from medical devices, and 28 (20%) were from inanimate surface environments in the ICU. Other 37 (20.5%) were no bacterial growth. A total of 238 bacterial isolates were identified. Out of 238 bacterial isolates, 133 (55.88%) were Gram-positive bacteria and the rest 105 (44.12%) were Gram-negative bacteria. Seventy (49%) swabbed samples had multibacterial contaminations, of which *S. aureus* plus CoNS accounted for 13 (18.6%), *S. aureus* plus *K. pneumoniae* accounted for 8 (11.4%), *S. aureus* plus *E. coli* accounted for 7 (10%), CoNS plus *K. pneumoniae* accounted for 6 (9%), and CoNS plus *E. coli* accounted for 5 (7%). CoNS 72 (54.14%) and *S. aureus* 61 (45.86%) were predominant Gram-positive bacteria isolated from the swab samples in ICU.

From the Gram-negative isolates, *E. coli* 41 (39%) were the most predominant bacteria followed by *K. pneumoniae* 30 (28.6%), *P. aeruginosa* 13 (12.4%), *A. baumannii* 11 (10.5%), *Enterobacter* spp. 6 (5.7%) and *Salmonella* spp. 4 (3.8%). The details of the isolates are shown in Table 1.

### 3.2. Proportions of Bacterial Isolates in ICU Environments

From the total 180 swab samples, the highest bacterial contamination rate was observed in inanimate surfaces 28 (87.5%) and medical devices 37 (86%), followed by patients 54 (79.4%) and HCPs (health care providers) 24 (65%). The positive rates of each study participants are summarized in Table 2.

### 3.3. Bacterial Profile against Different Medical Devices and Inanimate Surfaces, Patients, and Health Care Providers in ICU Environments

High bacterial contamination rates were observed from different medical devices and inanimate surfaces, patients, and health care providers. The highest bacterial contaminate and multibacterial isolates were identified from patient beds, sphygmomanometer, and stethoscope and body incubator. *S. aureus* 8 (31%) was the dominant isolate, followed by CoNS*, E. coli*, and *K. pneumoniae* each was 4 (15%) from contaminated patient beds. Ten multibacterial isolates were identified from patients' beds with 3 (30%) *S. aureus* plus *K. pneumoniae*, 2 (20%) *S. aureus* plus *E. coli*, 2 (20%) *S. aureus,* CoNS, and *A. baumannii* plus *Salmonella* spp., and CoNS plus *E. coli*, *S. aureus* plus CoNS, and *S. aureus,* and *K. pneumoniae* plus *Enterobacter* spp. multibacterial isolates each were 1 (10%). Multibacterial isolates 1 (25%) *(S. aureus* plus CoNS), (*S. aureus* plus *K. pneumoniae*), 1 (25%) (CoNS plus *K. pneumoniae*), and 1 (25%) (*S. aureus,* CoNS*, E. coli* plus *K. pneumoniae*) were identified from sphygmomanometer. Stethoscopes were contaminated with mixed isolates that include *S. aureus* plus CoNS, and *K. pneumoniae* plus *A. baumannii*. Also, multibacterial isolates were identified from body incubators which are *S. aureus* plus *K. pneumoniae*, CoNS plus *K. pneumoniae*, and CoNS plus *E. coli*.

A total of 105 bacterial contaminants were recovered from swabs collected from the hands of patients. Among isolates, 58 (55%) were Gram-positive and 47 (45%) were Gram-negative bacteria. From the Gram-positive isolates, CoNS 31 (53%) were predominant followed by *S. aureus* 27 (47%). *E. coli* 22 (47%) were the dominant isolate, followed by *K. pneumoniae* 16 (34%) from Gram-negative rods. Thirty-five (51%) out of the sixty-eight swabbed specimens had mixed growth. The predominant multibacterial isolates include *S. aureus* plus CoNS 7 (20%)*, S. aureus*, CoNS plus *E. coli*, and CoNS plus *K. pneumoniae* each was 4 (11.4%) and CoNS plus *E. coli* and *S. aureus* plus *K. pneumoniae* each was 3 (8.6%).

**Figure 1:**
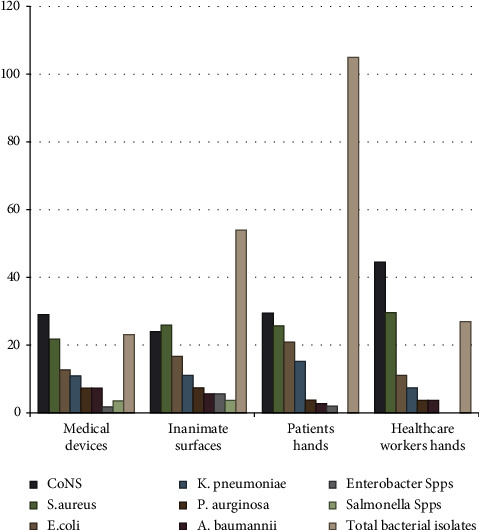
Distribution of bacterial profile at different medical devices and inanimate surfaces, patients, and health care providers' hands in ICU environments at Wachemo University Nigist Ellen Mohammed Memorial Comprehensive Specialized Hospital, August 1, 2022, to October 30, 2022.

A total of 27 bacterial isolates were identified from the hands of health care providers, 20 (74%) were Gram-positive bacteria and the rest 7 (26%) were Gram-negative bacteria. From the isolates, CoNS 12 (44.4%) was the most common dominant bacterial isolates. Four (15%) mixed bacterial isolates were identified that include *S. aureus* with *E. coli*, *S. aureus* with CoNS*, E. coli* with *K. pneumoniae*, and CoNS with *P. aeruginosa* that is shown in Figure 1.

### 3.4. Antimicrobial Susceptibility Patterns

The susceptibility patterns of isolates revealed varying degrees of resistance to the antibiotics tested. Gram-negative bacteria isolated from different sample sources were highly resistant to most of the antibiotics tested. From isolates, 94% were resistant to ampicillin, 87% to cotrimoxazole, 69% to cefoxitin, 68% to tetracycline, 66% to ceftriaxone, 62% to meropenem, and 59% to ceftazidime. In contrast, nitrofurantoin 76% and gentamicin 72% were the most effective antibiotics for all Gram- negative isolates, whereas Gram-positive bacterial isolates were found highly resistant to ampicillin and cotrimoxazole each 67%, cefoxitin 65%, and methicillin 60%,; however, they were mostly sensitive to vancomycin 83%, nitrofurantoin 86%, clindamycin and gentamicin each with 76%, and chloramphenicol 77%.

From Gram-negative isolate, *E. coli* demonstrated high level resistance to ampicillin (90.0%), amoxicillin/clavulanic acid (78.0%), ceftriaxone (76.0%), and cefoxitin (73%). Similarly, *K. pneumoniae, P. aeruginosa, A. baumannii*, and *Salmonella* species were highly resistant to cotrimoxazole and ampicillin each (100%). *K. pneumoniae* (97%), *P. aeruginosa* (92%), *A. baumannii (91%)*, and *Salmonella* species (100%) were resistant to tetracycline. CoNS 94% and *S. aureus* 75% were sensitive to nitrofurantoin (indicated in Table 3).

### 3.5. Multidrug-Resistant Pattern of Isolated Bacterial Pathogens

Of the total 238 bacterial isolates, 159 (66.8%) isolates were resistant to at least 3 antibiotics. Multidrug resistant (MDR) profiles were detected among 76.2% (80/105) of Gram-negative bacteria and 59.4% (79/133) of Gram-positive bacteria. The predominant MDR profile among Gram-negative bacteria was observed in *E. coli* (35/41, 85.4%), followed by *A*. *baumannii* (9/11, 82.2%), *K. pneumoniae* (22/30, 73.3%), *P. aeruginosa* (9/13, 62.2%), *Enterobacter* species (3/6, 50%), and *Salmonella* species (2/4, 50%). The most common MDR combinations found by *E. coli* and *K. pneumonia* were against 8 classes of antibiotics. Also, MDR combinations found by *E. coli*, *K. pneumoniae*, and *A*. *baumannii* were against 7 classes of antibiotics. The predominant MDR profile among Gram-positive bacteria was observed in *S. aureus* (40/61, 65.6%), followed by CoNS (39/72, 54.2%). The most common MDR combinations found by *S. aureus* and CoNS were against 8 and 7 classes of antibiotics (Table 4).

## 4. Discussion

This study was aimed to assess the bacterial profile from the intensive care unit at WUNEMMCSH (Wachemo University Nigest Ellen Mohammed Memorial Compressive Specialized Hospital), Southern Ethiopia, and evaluate their antimicrobial susceptibility patterns. Evidence-based knowledge about the extent of contamination of the hospital environment is important for designing and implementing effective prevention and control measure to tackle hospital-acquired infections. Moreover, the study results may give an insight for health professionals. While intensive care units are expected to be clean, they are usually reservoirs of common hospital-acquired bacterial pathogens and multidrug-resistant bacterial isolates. The output of this study showed that various intensive care unit environments were directly or indirectly contaminated by eight potential pathogenic bacterial isolates such as CoNS*, S. aureus, E. coli, Klebsiella pneumoniae, Pseudomonas aeruginosa, A. baumannii, Enterobacter*, and *Salmonella* spp. that raise serious concerns about possible nosocomial transmission. In addition, determination of antibiotic resistance of isolates from hospital settings was performed with particular emphasis on study groups in the intensive care unit.

In this study out of 180 swab, 143 (79.4%) of the various study swabs were contaminated with bacterial isolates, from which 86.7% of medical devices and inanimate surfaces were positive for at least one isolate. This is relatively in agreement with other study results, from medical devices and inanimate surfaces in Bahir Dar city 87.7% [20], Mekelle, Ethiopia 88.5% [27], and Zimbabwe 86.2% [4]. This finding contradicts with that of the studies conducted elsewhere due to lower bacterial contaminations observed in India being 54.4% [28], in Arba Minch 71.7% [29], and due to higher bacterial contaminations observed in studies conducted elsewhere in Windhoek, Namibia, which is 95% [30]. These deviations may be due to variation in study time and location, poor infection control and hand hygiene practices, intensive care unit room ventilation system, sterilization, procedures in disinfection, and sampling techniques. Similarly, in this study, the highest bacterial contaminate and multibacterial isolates were identified from patient beds, sphygmomanometer, stethoscope, body incubator, and others. This is in agreement with other studies conducted in Iran [3], Mekelle, Ethiopia [27], and Bahir Dar city [20]. Also, from the current study, 64.8% of health care providers' hands were contaminated with bacteria. Our result relatively agreed with that of other reports where bacterial contamination was found to be 64.5% in Iran [3]. It contrasts with current study, and lower bacterial contaminations were observed in India 17.8% [28] and Namibia 5% of HCP [30]. These variations may be due to wearing single personal protective gowns by many staff, investigating patients without gloves, poor infection prevention and hand-washing practice of patients, poor disinfection practice for medical devices and closely contacted inanimate patient surfaces before and after patient examinations, and higher bacterial isolates were identified from studies in Zimbabwe (100%) [31] due to unregular cleaning of hands with antiseptics and not disinfecting the most touched surfaces of medical equipment and inanimate surfaces. In this study, 79.4% of patients were contaminated with at least one bacterium isolates. It is relatively agreed with the study conducted in Tanzania 88.5% in patients [32].

The results of our study showed that varied groups of bacteria, including both Gram-positive (56%) and Gram-negative (44%) bacteria contaminated the ICU environments. Comparable to our results, the frequency of Gram-positive bacteria constituted the leading inhabited bacteria compared to Gram-negative bacteria in different countries, for example, in Iran (60.7% vs. 39.3%) [33], Nigeria (52.2% vs. 47.8%) [28], and Gondar in Ethiopia (60.5% vs. 39.5%) [34]. The dominance of Gram-positive over Gram-negative bacteria could be described by the fact that Gram-positive bacteria, being free of lipid-dominant drying up prone outer membrane, have concerning nature ability to keep their viability on different hospital environments for several time periods [33]. In contrast to our results, the study conducted from Addis Ababa in Ethiopia reported that Gram-negative bacteria were predominant than Gram-positive ones (82.6%, 17.4%) [10] and in Zimbabwe (66.2% vs. 33.82%, respectively) [4], and these variations may be due to different sampling times (endemic vs outbreak situations), the presence of colonized and/or infected patients during sampling, and the use of different sampling techniques and culture methodologies.

From Gram-positive isolates, CoNS (54.1%) was the most frequently identified bacteria, which were relatively similar to the findings of the previous studies conducted in Arba Minch 52.2% [29]. But it is higher than in the studies conducted in Mekelle 34.9% [27], India 10.7% [28], Uganda 6%, [35], and Mizan-Tepi 19.3% [36], and it is lower than in the studies conducted in Windhoek, Namibia 70% [30]. Similarly, *S. aureus* (46%) were the second most prevalent bacterial isolates which is in agreement with the finding of the study conducted in Arba Minch 47.7% [29]. But it is contradicted to the findings the studies conducted in Turkey 18.2% [37], Sudan 20% [38], Mekelle 26.3% [27], Mizan-Tepi 21.6% [36], Windhoek, Namibia 5% [30], and India 10.7% [28]. This recognized prevalence of GPB might be due to the bacterial resistance ability to dry conditions of the hospital environment and transmission from skin, nasal, and hands of health care providers and patients, and also the presence of underlying clinical conditions and immunocompromised patients admitted to intensive care.

From Gram-negative isolates, *E. coli* 41 (39%) was highly isolates from the all study groups in these output, but it contradicts with the findings of the study in Namibia 24% [30], Gondar 16.3%, [34], Madda-Walabu 20.9% [39], Mizan-Tepi 11.4% [36], Gondar [34], Northern Nigeria 8.0% [40], Mekelle 0.8%, [27] and Hiwot Fana 7.3% [41]. This could be explained by existence of colonized/infected patients in the ICU environments of the hospital, or failure of routine cleaning and disinfection practices. Also, *K. pneumoniae* (28.6%) was the second reported GNB, and it is relatively similar to the finding of a previous study conducted in Zimbabwe 20.3% [42]. But, it is higher than those of the studies carried out in Uganda 13% [35], Hiwot Fana 12.4% [41], Mizan-Tepi 14.8% [36], Namibia 3% [30], Northern Nigeria 2.4% [40], Mekelle 8% [27], and Gondar 4.08% [34]. This potential variation might be due to the dissemination of *K. pneumoniae* throughout the ICU environments during cleaning and recontamination of HCP hands with isolates during patient's examinations, hand-contact made with patients or inanimate surfaces, and hand-washing.

*P. aeruginosa* (12.4%) was the third-reported GNB, and it was almost similar to the findings of the study conducted in Hiwot Fana hospital, Ethiopia 7.3% [41], Gondar, Ethiopia 11.4% [34], and Mizan-Tepi, Ethiopia 11.4% [36]. It contradicts with that of the study in conducted in Mekelle 1.3% [27]. This difference might be due to the primary distribution of microorganism to hospital environment, and it is also particularly well-adapted to live in wet or moist conditions. In this study, 10.5% *Acinetobacter baumannii* was identified. Comparable results were found in studies conducted in Addis Ababa, Ethiopia, in which 17.4% of *Acinetobacter baumannii* were found [10], and in Iran (8.3%) [43] and Windhoek, Namibia, (5.3%) [30]. *Salmonella* species 4 (3.8%) is potentially pathogenic isolates which was identified from medical devices and inanimate surfaces. It is similar with the findings of the study conducted in Mekelle (3.5%) [27] and Hiwot Fana (7.3%) [41], but it is lower than that of the study conducted in Arba Minch (23.3%) [29]. The presence of these microorganisms might be due to the aspiration of secretions in colonized patients in the unit or poor hand hygiene practices and contaminated equipment/inanimate surface.

In these studies, most of the Gram-negative bacteria were resistant to most of the tested antibiotics such as ampicillin (94%), cotrimoxazole (87%), ceftriaxone (69%), amoxicillin/clavulanic acid (75.0%), cefoxitin (69%), tetracycline (68%), and ceftazidime (59%). The high levels of resistance to these antibiotics were associated with the antibiotics that are most frequently used in empirical, and serious problems can be encountered while prescribing those antibiotics. Providing updated information through guidelines for prescribing antibiotics becomes a necessity. Our results were comparable with those of the studies conducted elsewhere such as from Zimbabwe [4] (ampicillin 80% to 84.6%), India [28] (cotrimoxazole 83.3% and ceftazidime 83.3%), India [3] (cefoxitin 57% to 100%), and Windhoek, Namibia [35] and Mekelle (ampicillin 82%) [27]. In this study, ciprofloxacin (48%) and gentamycin (76%) were the most effective antibiotics for all Gram-negative isolates, and this finding is similar to that of the study conducted in Mekelle (ciprofloxacillin 87% and gentamicin 91.4%) [33] but is sensitive to cefoxitin (78%) in Hiwot Fana hospital [41].

In this study, *E. coli* 39% and *P. aeruginosa* 92% were resistant to tetracycline, which is in line with similar resistance rates *E. coli* 50% and *P. aeruginosa* 83.3% from the study conducted in Hawassa [44]. Also, in these studies, vancomycin (83%) was the most sensitive tested antibiotic for Gram-positive bacterial isolates, which is similar to that of the study conducted in Zimbabwe (78%) [4] but contradicts with that of the study for resistance to vancomycin (100% in India [3]. CoNS and *S. aureus* were mostly resistant to ampicillin (67%), cloxacillin (59%), and cotrimoxazole (67%) which was similar to the findings of the studies conducted in Nigeria [28] and Bahir Dar [45]. Also, in this study, *S*. *aureus* was resistant to cotrimoxazole (69%) and ciprofloxacin (54%) but most sensitive to gentamicin (76%). This finding is relatively similar with resistance to cotrimoxazole (79%) and ciprofloxacin (50%) but sensitive to gentamycin (96%) in the study in Sudan [38]. Our results showed that 159 (66.8%) bacterial isolates were resistant to at least 3 antibiotics. Multidrug resistance was detected both in 56.4% of Gram-positive and 76.2% of Gram-negative isolates, which was in line with the findings of other studies, which were conducted in Sudan (84.5%) [38], Bahir (57.5%) [20], and Hawassa (57.7%) [44].

### 4.1. Limitation of the Study

The study has a number of advantages. It gives useful information about the bacterial profile and antimicrobial susceptibility trends of potential pathogenic bacteria in hospital settings, especially in ICU rooms. However, the study does have the following drawbacks:

- It was carried out in a single hospital, which might not be an accurate representation of other hospitals in the area
- Anaerobic and/or fastidiously growing bacteria in this study were not investigated because the isolation of these requires special procedures and equipment that were not available
- Other microbial contaminants, such as parasites, virus, molds, and yeasts, which were beyond the scope of this study, were not investigated
- Moreover, settle plate samples were not taken from the ICU environment of the hospitals and samples were taken once from each selected study participants

## 5. Conclusion and Recommendations

The inanimate hospital environments, medical device, health care providers, and patients in the ICU rooms of WUNEMMCSH (Wachemo University Nigest Ellen Mohammed Memorial Comprehensive Specialized Hospital) were colonized with 143 (79.4%) potential pathogenic bacterial isolates, which can cause nosocomial infections with high rates of morbidity and mortality among patients. CoNS were the predominant bacterial type, followed by *S. aureus and E. coli*. Nitrofurantoin was the most sensitive drug for all isolates; similarly gentamicin and vancomycin was the most effective antibiotics for all Gram-negative and Gram-positive isolates, respectively. The frequency of MDR bacterial pathogens was found to be 159 (66.8%), which was alarmingly high. This might be a reflection of inappropriate use of antibiotics or unavailability of a guideline regarding the selection of drugs. The presence of MDR in ICU environments may be a predisposing factor for infection. Therefore, to reduce the risk of bacterial contamination and MDR, strict adherence to hospital infection prevention and control measures should be enforced. These measures include performing regularly hand hygiene, periodic disinfection, and sterilization of medical equipment.

## Figures and Tables

**Table 1:** Bacterial isolates from ICU environments at Wachemo University Nigist Ellen Mohammed Memorial Comprehensive Specialized Hospital, August 1, 2022, to October 30, 2022.

Types of bacterial isolated from ICU	Number	Percent (%)	95% CI
Gram-negative bacteria isolates	105	44.12	37.7–50.3
*E. coli*	41	39	29.7–48.3
*K. pneumoniae*	30	28.6	20–37.2
*P. aeruginosa*	13	12.4	6.1–18.7
*A. baumannii*	11	10.5	4.6–16.4
*Enterobacter* species	6	5.7	1.3–10.1
*Salmonella* species	4	3.8	0.1–7.5

Gram-positive bacteria isolates	133	55.88	49.7–62.3
CoNS	72	54.14	45.6–62.6
*S. aureus*	61	45.86	37.5–54.5
Total	238	100	

CoNS: coagulase-negative staphylococci; CI: confidence interval.

**Table 2:** Proportion of bacterial isolates from different study groups in ICU environments at Wachemo University Nigist Ellen Mohammed Memorial Comprehensive Specialized Hospital, August 1, 2022, to October 30, 2022

Study groups screened	Positive rate *N* (%)	95% confidence interval
Medical devices (*n* = 45)	37 (86)	80.3–91.7
Inanimate surfaces (*n* = 32)	28 (87.5)	82.1–92.9
Patients (*n* = 68)	54 (79.4)	72.8–92.9
Health care providers (*n* = 37)	24 (65)	57.2–72.8
Total (*n* = 180)	143 (79.4)	72.8–92.9

**Table 3:** Antimicrobial resistance profile of bacterial isolates from ICU environments at Wachemo University Nigist Ellen Mohammed Memorial Comprehensive Specialized Hospital, August 1, 2022, to October 30, 2022

Bacterial isolates	Antimicrobials tested for Gram-negative isolates												
	NIT	CIP	CXT	CAZ	AMC	CN	CHL	CXM	MER	CTR	COT	AMP	TE
*E. coli* 41 (39%)	5 (12.2%)	15 (37%)	30 (73%)	27 (66%)	32 (78%)	18 (44%)	21 (51%)	19 (46%)	25 (61%)	31 (76%)	29 (71%)	37 (90%)	16 (39%)
*K. pneumoniae* 30 (28.6%)	7 (23.3%)	18 (60%)	23 (77%)	13 (43%)	16 (53%)	10 (33%)	10 (33%)	12 (40%)	22 (73%)	17 (57%)	30 (100%)	30 (100%)	29 (97%)
*P. aeruginosa 13 (12.4%)*	5 (38%)	6 (46%)	7 (54%)	9 (69%)	10 (77%)	5 (38%)	10 (77%)	5 (38%)	8 (62%)	8 (62%)	13 (100%)	13 (100%)	12 (92%)
*A. baumannii* 11 (10.5%)	5 (45%)	6 (55%)	8 (73%)	7 (64%)	11 (100%)	3 (27%)	2 (18%)	8 (73%)	3 (27%)	8 (73%)	11 (100%)	11 (100%)	10 (91%)
*Enterobacter* species 6 (5.7%)	2 (33%)	4 (67%)	2 (33%)	4 (67%)	5 (83%)	1 (17%)	3 (50%)	0 (0%)	4 (67%)	3 (50%)	4 (67%)	4 (67%)	0 (0%)
*Salmonella* species 4 (3.8%)	1 (25%)	1 (25%)	2 (50%)	2 (50%)	3 (75%)	2 (50%)	0 (0%)	0 (0%)	3 (75%)	2 (50%)	4 (100%)	4 (100%)	4 (100%)
Total *n* = 105	25 (24%)	50 (48%)	72 (69%)	62 (59%)	45 (43%)	29 (28%)	45 (43%)	44 (42%)	65 (62%)	69 (66%)	91 (87%)	99 (94%)	71 (68%)

CoNS 72 (54%)	4 (6%)	29 (40%)	44 (61%)	16 (22%)	12 (17%)	20 (28%)	18 (25%)	31 (43%)	38 (53%)	46 (64%)	47 (65%)	48 (67%)	27 (38%)
*S. aureus* 61 (46%)	15 (25%)	33 (54%)	42 (69%)	16 (26%)	11 (18%)	12 (20%)	13 (21%)	24 (39%)	41 (67%)	34 (56%)	42 (69%)	41 (67%)	24 (39%)
Total (*n* = 133)	19 (14%)	62 (47%)	86 (65%)	32 (24%)	23 (17%)	32 (24%)	31 (23%)	55 (41%)	79 (59%)	80 (60%)	89 (67%)	89 (67%)	51 (38%)

AMC = amoxicillin/clavulanic acid, AMP = ampicillin, CHL = chloramphenicol, CAZ = ceftazidime, CIP = ciprofloxacin, CoNS = coagulase-negative *Staphylococcus*, CTR = ceftriaxone, CLN = clindamycin, AZM = azithromycin, CN = gentamicin, CXM = cefixime, MER = meropenem, NIT =  nitrofurantoin, MET =  methicillin, COT =  cotrimoxazole TE = tetracycline, VAN = vancomycin, CX = cloxacillin, and CXT =  cefoxitin.

**Table 4:** Multidrug-resistance pattern of bacterial isolates from ICU environments at Wachemo University Nigist Ellen Mohammed Memorial Comprehensive Specialized Hospital, August 1, 2022, to October 30, 2022.

Multidrug-resistance profiles of Gram-negative isolates	
Bacterial isolates and MDR profiles	Frequency *n* (%)
*E. coli*	
NIT, CIP, CXT, CN, MER, CTR, COT, AMP	2 (4.9%)
CIP, CAZ, AMC, CHL, CXM, MER, COT	2 (4.9%)
CXT, CAZ, AMC, CN, CHL, COT, AMP	1 (2.4%)
CIP, CXT, CAZ, MER, COT, AMP	3 (7.3%)
CAZ, AMC, CN, CHL, CTR, AMP	2 (4.9%)
NIT, CXT, CTR, COT, AMP, TE	1 (2.4%)
CXT, MER, CTR, COT, AMP	4 (9.8%)
CAZ, AMC, CHL, MER, COT	2 (4.9%)
CIP, CAZ, CXM, CTR, COT	2 (4.9%)
CXT, CN, MER, CTR, TE	1 (2.4%)
CXT, CAZ, MER, CTR	6 (14.6%)
CAZ, CN, CHL, AMP	5 (12.2%)
CIP, CTR, AMP	4 (9.8%)
Total	35/41 (85.4%)

*K. pneumoniae*	
NIT, CIP, CXT, AMC, MER, CTR, COT, AMP	4 (13.4%)
CIP, CXT, CAZ, CN, CHL, COT, AMP	2 (6.7%)
CIP, AMC, MER, CTR, COT, AMP, TE	2 (6.7%)
NIT, CAZ, CN, CHL, COT, AMP	1 (3.35%)
CAZ, AMC, CXM, COT, AMP	1 (3.35%)
CXT, MER, AMP, TE	6 (20%)
AMC, CXM, MER, COT	3 (10%)
CIP, COT, TE	3 (10%)
Total	22/30 (73.3%)

*P. aeruginosa*	
CIP, CAZ, AMC, CHL, MER, TE	4 (30.8%)
NIT, CXT, CN, COT, AMP	2 (15.4%)
CAZ, AMC, CTR, AMP, TE	1 (7.7%)
CHL, CTR, COT	1 (7.7%)
CIP, CXT, MER	1 (7.7%)
Total	9/13 (62.2%)

*A. baumannii*	
AMC, CAZ, CXM, CTR, COT, AMP, TE	3 (27.3%)
CIP, CXT, AMC, MER	2 (18.2%)
NIT, CAZ, CTR, COT	2 (18.2%)
CXT, CN, CTR	1 (9.1%)
COT, AMP, TE	1 (9.1%)
Total	9/11 (82.2%)

*Enterobacter* species	
CIP, CAZ, AMC	1 (16.7%)
CHL, MER, COT	1 (16.7%)
AMC, CTR, AMP	1 (16.7%)
Total	3/6 (50%)

*Salmonella* species	
CIP, AMC, CTR, COT	1 (25%)
MER, AMP, TE	1 (25%)
Total	2/4 (50%)

CoNS	
CIP, CXT, CLN, AZM, MET, CLX, COT, TE	2 (%)
NIT, CIP, VAN, CN, CHL, CLX, COT, AMP	1 (1.4%)
CIP, CXT, CN, AZM, MET, COT, TE	3 (4.2%)
CXT, CHL, CLX, COT, AMP	7 (9.7%)
CIP, CN, CLX, AMP	7 (9.7%)
CXT, CLN, VAN, AZM	4 (5.6%)
CLX, COT, AMP	9 (9.7%)
CIP, CXT, TE	6 (8.3%)
Total	39/72 (54.2%)

*S. aureus*	
NIT, CIP, CXT, AZM, CLX, MET, COT, AMP	4 (6.6%)
CIP, CXT, CLN, VAN, CN, CHL, AZM	2 (3.3%)
NIT, CIP, CN, CHL, COT, AMP, TE	1 (1.6%)
CXT, CLN, CLX, MET, COT, TE	3 (4.9%)
NIT, CIP, CXT, VAN, CN, TE	2 (3.3%)
CXT, AZM, CLX, COT, AMP	6 (9.8%)
CIP, CXT, COT, AMP	6 (9.8%)
NIT, CLX, MET, TE	4 (6.6%)
CIP, COT, AMP	9 (14.8%)
CLN, CN, MET	3 (4.9%)
Total	40/61 (65.6%)

Total MDR isolates	159/238 (66.8%), (95% CI = (60.8, 72.78))

## Data Availability

All relevant data are within the article, and additional data are available from the corresponding author upon request.
